# PathInHydro, a Set of Machine Learning Models to Identify Unbinding Pathways of Gas Molecules in [NiFe] Hydrogenases

**DOI:** 10.1021/acs.jcim.4c01656

**Published:** 2025-01-07

**Authors:** Farzin Sohraby, Jing-Yao Guo, Ariane Nunes-Alves

**Affiliations:** Institute of Chemistry, Technische Universität Berlin, Straße des 17. Juni 135, Berlin 10623, Germany

## Abstract

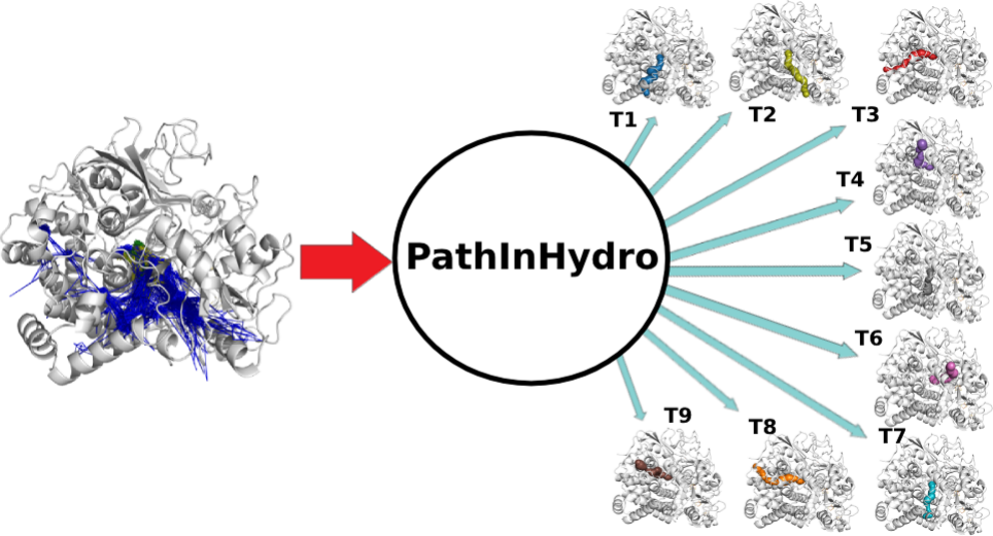

Machine learning (ML) is a powerful tool for the automated data analysis of molecular dynamics (MD) simulations. Recent studies showed that ML models can be used to identify protein–ligand unbinding pathways and understand the underlying mechanism. To expedite the examination of MD simulations, we constructed PathInHydro, a set of supervised ML models capable of automatically assigning unbinding pathways for the dissociation of gas molecules from [NiFe] hydrogenases, using the unbinding trajectories of CO and H_2_ from*Desulfovibrio fructosovorans* [NiFe] hydrogenase as a training set. [NiFe] hydrogenases are receiving increasing attention in biotechnology due to their high efficiency in the generation of H_2_, which is considered by many to be the fuel of the future. However, some of these enzymes are sensitive to O_2_ and CO. Many efforts have been made to rectify this problem and generate air-stable enzymes by introducing mutations that selectively regulate the access of specific gas molecules to the catalytic site. Herein, we showcase the performance of PathInHydro for the identification of unbinding paths in different test sets, including another gas molecule and a different [NiFe] hydrogenase, which demonstrates its feasibility for the trajectory analysis of a diversity of gas molecules along enzymes with mutations and sequence differences. PathInHydro allows the user to skip time-consuming manual analysis and visual inspection, facilitating data analysis for MD simulations of ligand unbinding from [NiFe] hydrogenases. The codes and data sets are available online: https://github.com/FarzinSohraby/PathInHydro.

## Introduction

Pathways for the binding of ligands and gas molecules to enzymes and proteins can be exploited in biotechnology, enzyme engineering and drug design to improve catalysis and drug efficacy.^1−3^ In biotechnology, mutant enzymes can be rationally designed to block or promote access of substrate or inhibitor molecules to the catalytic site. For example, previous work introduced mutations in [NiFe] hydrogenase in an attempt to block the access of inhibitor molecules such as O_2_ and CO to the catalytic site.^4^ The mutations resulted in slower inhibitor binding, but no inhibitor resistance. Another work proposed mutations in the enzyme epoxide hydrolase to facilitate product release.^5^ Such mutations led to increased enzymatic catalytic activity. In drug design, pathways for ligand unbinding are associated with protein–ligand residence times, which can be better correlated with drug efficacy than thermodynamic parameters.^2^ While it is usually not possible to observe ligand unbinding paths in experiments, such paths have been extensively investigated using molecular dynamics (MD) simulations.^2,3,6−13^

Machine Learning (ML) has become an imperative tool in the field of computational biochemistry to analyze MD simulations, facilitating the recognition of patterns and trends, identifying subtle details that can escape intuition-based manual assessments, and making it easy to analyze a large number of trajectories. In recent years, various ML methods have been developed and utilized to identify binding pathways in MD simulations.^14−22^ Ray and Parrinello^14^ reported a method based on the dynamic time-warping (DTW) algorithm^23^ to align trajectories, in combination with K-medoid clustering, to delineate binding pathways based on distances or contacts between the ligand and the protein residues. Motta et al.^15^ developed a pathway recognition tool called PathDetect-SOM, based on the deep learning algorithm self-organizing maps (SOM), to cluster and classify ligand (un)binding pathways. Kokh et al.^16^ proposed a workflow to identify ligand unbinding paths using fingerprints of protein–ligand interactions and hierarchical clustering. This workflow was used to identify ligand unbinding paths for T4 lysozyme mutants.^13^ Bray and co-workers^17^ presented two methods for ligand unbinding pathway classification. One of the methods uses principal component analysis (PCA) of protein–ligand contacts (conPCA)^24,25^ in combination with gradient boosting (PCA-ML). The other method is RMSD-based clustering of the trajectories, which the authors considered more reliable, because it takes ligand conformational changes into account. Expanding on this work, Tänzel et al.^18^ reported a novel ML technique where unbinding trajectories are clustered using the Leiden community detection algorithm, which uses a similarity measure—based on either the RMSD or the conPCA in the benchmark tests of the authors—in combination with a resolution parameter, for dissociation pathway classification. A popular software tool is AQUA-DUCT,^26^ which is suitable to identify unbinding paths in proteins with buried binding sites. AQUA-DUCT tracks the motion of ligands within proteins, and trajectories can be clustered using different algorithms. Additionally, Sarkar et al.^22^ recently developed a protocol where tunnel utilization was determined based on whether the ligand passed the tunnel bottleneck in the trajectory. Overall, it is clear that, with the increase in computational power dedicated to MD simulations for the analysis of ligand binding kinetic rates and populations of ligand binding paths, manual examination of the trajectories from MD simulations is becoming less feasible, while automated tools and methods are gaining traction.

**Figure 1:**
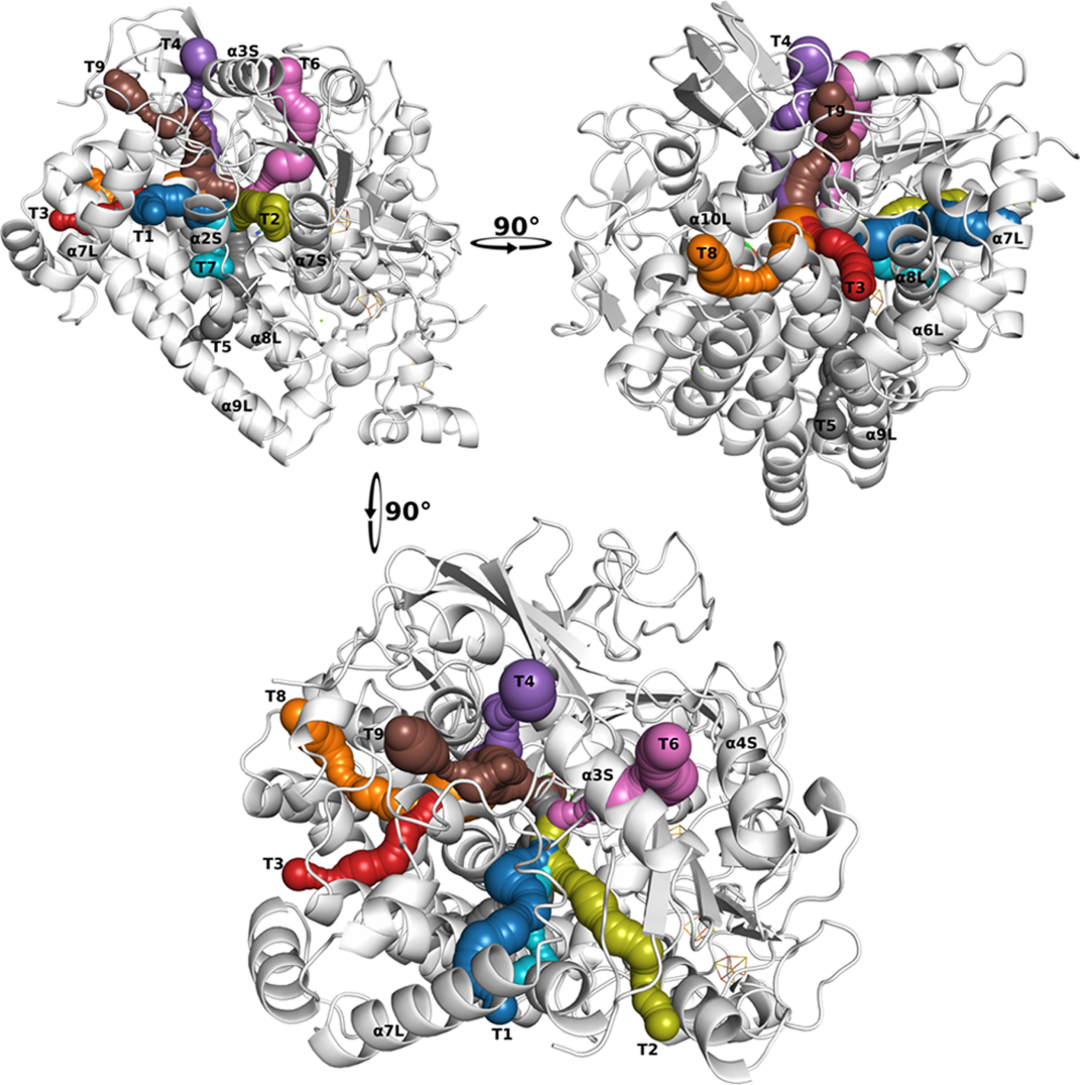
Unbinding pathways of CO from the H_2_ase from*Desulfovibrio fructosovorans* (crystallographic structure from PDB ID 1YQW(40)). A total of 9 tunnels (T1-T9) were identified in the crystallographic structure by the CAVER 3.0 plugin in Pymol^26,39^ as detailed in an earlier work,^6^ and they correspond to CO unbinding paths identified in MD simulations in the same work.^6^ The secondary structural elements are named based on the order in the protein sequence and location in the Small (S) or Large (L) subunits. For example, α7S means the seventh α-helix in the small subunit.

[NiFe] hydrogenase (H_2_ase) is a member of the hydrogenase family which has the ability to oxidize hydrogen molecules and produce electricity or vice versa (H_2_ ⇌ 2 H^+^ + 2 e^–^).^27,28^ The high catalytic activity of this enzyme makes it an attractive candidate to be used in biofuel production pipelines.^29−31^ However, some of these enzymes evolved in anaerobic organisms, and as a result are air-sensitive. Common gas molecules present in the atmosphere such as O_2_ can destroy the catalytic site or heavily inhibit the enzyme.^32−34^ Since the catalytic site is located at the center of the enzyme, connected to the surface by tunnels that can reach a length of roughly 2 to 3 nm (Figure 1), a promising strategy to overcome inhibition is to introduce mutations in the tunnels to block the access of inhibitor gas molecules to the catalytic site, while the small hydrogen molecules can still enter unhindered.^35−37^

In previous works, this approach has been applied to the H_2_ase from *Desulfovibrio fructosovorans* (Df H_2_ase), where mutations were introduced to block the access of the inhibitors CO and O_2_ to the catalytic site. Liebgott et al.^4^ evaluated the effects of 10 different mutations to the residues at positions 74 and 122 in the vicinity of the catalytic site, by experimentally measuring the (un)binding kinetic rates of both CO and O_2_. While some mutations reduced the association rate constant for the binding of inhibitors to the Df H_2_ase, none of the mutations resulted in an inhibitor-resistant enzyme. In a recent study, we used τ-random accelerated molecular dynamics (τRAMD)^38^ to simulate unbinding events, and analyzed in detail the unbinding of CO from the wild type (WT) and the 10 different mutants of Df H_2_ase.^6^ The computed relative residence times were in agreement with the experimental results. Additionally, we simulated the unbinding of H_2_ and O_2_ from the WT Df H_2_ase, and detected different secondary unbinding paths for the substrate and inhibitors, which led us to propose different sites of mutations to improve the air resistance of Df H_2_ase. In our previous work, we identified 9 different tunnels connecting the catalytic site to the surface of the enzyme using CAVER 3.0.^39^ The tunnels were associated with unbinding paths from τRAMD trajectories, identified using AQUA-DUCT 1.0^26^ and mean shift for clustering. While AQUA-DUCT effectively facilitated the identification of unbinding paths, some manual labor and visual inspection were required to compare and match paths for different mutants, and to match the paths with the tunnels identified.

In this work, utilizing a data set of trajectories from MD simulations for the unbinding of different gas molecules from Df H_2_ase previously obtained using τRAMD,^6^ we built PathInHydro, a set of supervised ML models that are able to identify unbinding pathways in trajectories, using as features the ligand contacts with the enzyme residues. We trained the models using only a portion of the data containing CO and H_2_’s unbinding trajectories from Df H_2_ase, and subsequently tested the models on the unbinding trajectories of O_2_ from Df H_2_ase, as well as CO, H_2_ and O_2_ unbinding from*Megalodesulfovibrio gigas* H_2_ase (Mdg H_2_ase). PathInHydro can help streamline the examination of simulations of H_2_ase mutants in the search for inhibitor-tolerant enzymes, allowing one to skip the manual effort and time-consuming visual inspection of the trajectories for assigning unbinding pathways.

## Methods

### Data Set

Two models were built: one binary model separating the pathways regularly traveled in Df H_2_ase and with high probabilities (T1, T2 and T7) from the ones that see less traffic and display lower probabilities (T3-T6, T8 and T9), and a multiclass model which aimed to distinguish each of the nine individual pathways (T1-T9).

The data used for the ML model construction were a collection of simulated unbinding trajectories of H_2_, CO and O_2_ produced using τRAMD, a large portion of which have been reported in our previous publication.^6^ The training data set consisted of CO unbinding trajectories from WT and 10 different mutants of Df H_2_ase, together with H_2_ unbinding trajectories from WT Df H_2_ase; for each of the 11 forms of the enzyme, 75 simulations were performed, resulting in a total of 825 unbinding events for CO and 74 unbinding events for H_2_. In the case of the binary model, we split the combined CO-and-H_2_ data set into train/validation/test sets with a 70:15:15 ratio, where the validation set is used for performance testing during algorithm selection and hyperparameter tuning. A stratified approach was adopted, where the ratio of CO and H_2_ data entries was kept consistent across the sets (each make up 92 and 8% of the data set, respectively). However, for the multiclass model, where the class imbalance issue is more extreme (see results), with less than 10 occurrences of the least populated paths, creating a validation set where the minority classes are extremely ill-represented would make the results unreliable. Therefore, we instead implemented a 70:30 train/test split for the combined CO and H_2_ unbinding trajectories, and utilized cross-validation for hyperparameter tuning.

Additionally, the unbinding trajectories from the WT enzyme for O_2_ as ligand were applied as an external test set, with 75 unbinding events collected. Furthermore, to examine the model’s generalizability toward more drastic sequence differences, we employed another member of the H_2_ase family, Mdg H_2_ase, to create an external test set. Using the EMBOSS Needle software^41^ and blosum62 scoring matrix^42^ to align and compare the sequences of the two enzymes, we discovered the similarity and identity measures to be 76.3 and 65.5%, respectively. The two enzymes were structurally aligned using UCSF chimera,^43^ and the backbone root-mean-square deviation (RMSD) value obtained was 0.6 Å. More details about the structure comparison between the two enzymes can be found in the Supporting Information (Figures S1–S3). We performed a new set of τRAMD simulations with the same procedure and parameters used in our previous study (details below) to acquire 75 unbinding events with the new type of enzyme and CO, H_2_ or O_2_ as ligands. The full distribution of data points into training, validation and test sets for the binary and multiclass models is provided in Tables S1 and S2.

### Features

We used as features the fraction of time in which the gas molecule was in contact with each enzyme residue during the trajectory. A contact was present when the distance between one atom in the gas molecule and one atom in the residue was equal to or lower than 4 Å. The contacts between the gas molecules and each residue of the enzyme were calculated with MDAnalysis.^44,45^ The matching of the residues for the two different enzymes was performed using the structure alignment provided by UCSF Chimera (Figures S2 and S3), with the sequence of the Df H_2_ase considered as the reference. The positions of the residues which were present in the Df H_2_ase but not in the Mdg H_2_ase (8–14, 153–154, 302–305, 342 in the large subunit, and 67–68 and 267 in the small subunit) were added to the features of the Mdg H_2_ase data set with the values set to zero; conversely, the positions of the residues that were only present in the Mdg H_2_ase (453 in the large subunit and 136–138 in the small subunit) were not included in the analysis.

For feature extraction, we excluded the section of the trajectories before the gas molecule escapes the bottleneck between residues 74 and 122.^6,46,47^ Before the gas molecule leaves this bottleneck, it is close to the catalytic site and can be considered as remaining in the bound state. Removal of this section significantly reduced the amount of noise in the data, and led to perceptible improvements in the ML models’ performances.

In the final data set, each trajectory contained 804 features, corresponding to the 804 shared residues in the enzymes, and the unbinding pathway (T1-T9), previously assigned using Caver and AQUA-DUCT, was set as the target variable.

To assess the information content of this feature set, we applied the t-distributed stochastic neighbor embedding (t-SNE) algorithm^48^ to each of the data sets, utilizing the function provided by the scikit-learn python library.^49^ This approach maps the high-dimensional feature set into the 2D space in an unsupervised manner, with the purpose of clustering neighboring entries and creating a clear visual representation to illustrate the data distribution.

### Machine Learning Algorithms

We used an algorithm selection procedure in which classification models were developed and optimized with multiple ML algorithms, and the algorithm with the overall best validation performance was selected for each task. The algorithms tested included random forest (RF), support vector machine, logistic regression, gradient boosting, adaptive boosting and *k*-nearest neighbors, as implemented in the scikit-learn python library.^49^ Adaptive boosting and RF were chosen for the binary and multiclass models, respectively (more information in Tables S3 and S4). An oversampling technique (the RandomOverSampler implemented in the imbalanced-learn library^50^) was applied to even out the imbalanced data distribution across different pathways. Hyperparameter tuning was performed using the GridsearchCV method from scikit-learn. We fixed the number of trees (n_estimators) to 500, and tested a variety of parameter combinations to regulate the model complexity, and adopted the combinations which allowed for a moderate level of regularization, while providing statistical measurements on par with the best-performing unregularized models. The hyperparameter values of the final models are as follows: n_estimators = 500 and learning_rate = 0.05 for the binary model using adaptive boosting; n_estimators = 500, ccp_alpha = 0.001, max_samples = 0.9 and max_depth = 30 for the multiclass model using RF. During the entire process of the ML model training and evaluation, we used random_state = 1. A baseline model, where all data entries were assigned to the dominant class, was built using the DummyClassifier method with the “most_frequent” strategy, as available in scikit-learn.^49^

### Evaluation of the Machine Learning Models

Receiver operating characteristic (ROC) curves (false positive rate, eq 1, vs true positive rate, eq 2, for classifications using different thresholds) were used to evaluate binary models. Balanced accuracy (BA), eq 3, and Matthews correlation coefficient (MCC), eq 4, were used as metrics to assess the performance of the binary and multiclass models while accounting for the heavy data imbalance.
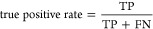
1

In the true positive rate equation, TP is the number of true positives and FN is the number of false negatives.
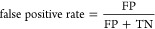
2

In the false positive rate equation, FP is the number of false positives and TN is the number of true negatives.
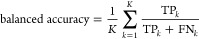
3

In the balanced accuracy equation, *K* is the number of classes, TP*_k_* is the number of true positives for class k, and FN*_k_* is the number of false negatives for class *k*.
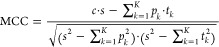
4

In the MCC equation, *k* is the class (from 1 to *K*), *s* is the number of samples, *c* is the number of samples correctly predicted, *t_k_* is the number of times class *k* truly occurred, *p_k_* is the number of times class k was predicted.

### Molecular Dynamics Simulations

We acquired the simulations of CO, H_2_ and O_2_ unbinding from Df H_2_ase in our previous work.^6^ The simulations of CO, H_2_ and O_2_ unbinding from Mdg H_2_ase were performed for this project using τRAMD and a methodology identical to the one previously employed to simulate the unbinding of gas molecules from Df H_2_ase. The crystal structure of Mdg H_2_ase was retrieved from the Protein Data Bank^51^ (PDB ID 1YQ9(40)). The protonation states of the residues at pH 7 were determined using Propka version 3.5.2,^52−54^ as implemented in the program pdb2pqr 2.1.1.^55,56^ The force field bonded parameters and the partial charges of the [NiFe] and [FeS] metal centers were gathered from the works of Smith et al.^57^ and Teixeira et al.,^58^ respectively. The partial charges for the atoms in the CO molecule were +0.059 e for C and −0.059 e for O, following quantum mechanical calculations performed in our previous studies.^6^ The bonded and Lennard-Jones parameters for CO were obtained from a publication by Straub and co-workers,^59^ and the bonded and Lennard-Jones parameters for H_2_ and O_2_ were obtained from the work of Wang et al.^60^

Ligand unbinding is a rare event which conventional MD simulations are often incapable of capturing. Therefore, we used an enhanced sampling method, τRAMD, to promote the occurrence of such an event. The τRAMD simulations of CO, H_2_ and O_2_ unbinding from Mdg H_2_ase were performed using GROMACS-RAMD version 2.0^16,38^ and the AMBER99SB force field^61^ to describe the system. The protein–ligand complex was centered in a simulation box with a 1 nm distance between the solute and the box edge, and then solvated with the TIP3P^62^ water model and 118 mM of sodium and chloride ions. The starting structure underwent energy minimization using the steepest descent algorithm until the maximum force was less than 10 kJ·mol^–1^·nm^–1^. The system was then heated to 310 K using the Berendsen thermostat.^63^ Subsequently, the pressure was equilibrated to 1 bar using the Berendsen barostat.^63^ Following temperature and pressure equilibration, positional restraints on the system’s heavy atoms were gradually reduced in four steps (500, 100, 10, and 0 kJ·mol^–1^·nm^–2^), while maintaining 5000 kJ·mol^–1^·nm^–2^ positional restraints on the ligand atoms in all simulations except the τRAMD unbinding runs. Postequilibration, temperature and pressure coupling were controlled by the Nose-Hoover^64,65^ thermostat and the Parrinello-Rahman barostat,^66,67^ respectively. Covalent bonds to hydrogen atoms were constrained using the linear constraint solver (LINCS) algorithm,^68^ and solvent bond lengths were constrained using the SETTLE algorithm.^69^ Long-range electrostatic interactions were handled using the particle mesh Ewald (PME) method with a real-space cutoff of 1.2 nm, a PME order of four, and a Fourier grid spacing of 1.2 Å.^70,71^ A time step of 2 fs was used. van der Waals forces were calculated with a cutoff of 1.2 nm. In order to follow the same procedure carried out for the Df H_2_ase, after equilibration we first performed one 50 ns conventional MD simulation, followed by five replicas of 20 ns conventional MD simulation to generate variability in starting configurations.

For the τRAMD runs, the force magnitude was set to 1 kcal·mol^–1^·Å^–1^, the threshold distance to 0.0025 nm and the evaluation frequency to 100 fs. Fifteen unbinding simulations were performed for each of the five replicas, resulting in a total of 75 unbinding events. Trajectories were assigned to tunnels through the combined use of AQUA-DUCT and visual inspection. Initially, AQUA-DUCT was employed to map the pathways of the gas molecule within the tunnels across 75 unbinding trajectories, subsequently clustering them. This clustering utilized the mean shift algorithm with a bandwidth of 7, while other settings remained at their default values. Following this, we visually analyzed the clusters of pathways, including their traces and exit points, then matched each cluster to one of the tunnels identified by CAVER in the crystal structure of Df H_2_ase. All of the parameters and steps done for these simulations and for the analysis were identical to the simulations and analysis performed in our related work using Df H_2_ase.^6^

## Results

### Distribution of the Data Set

**Figure 2:**
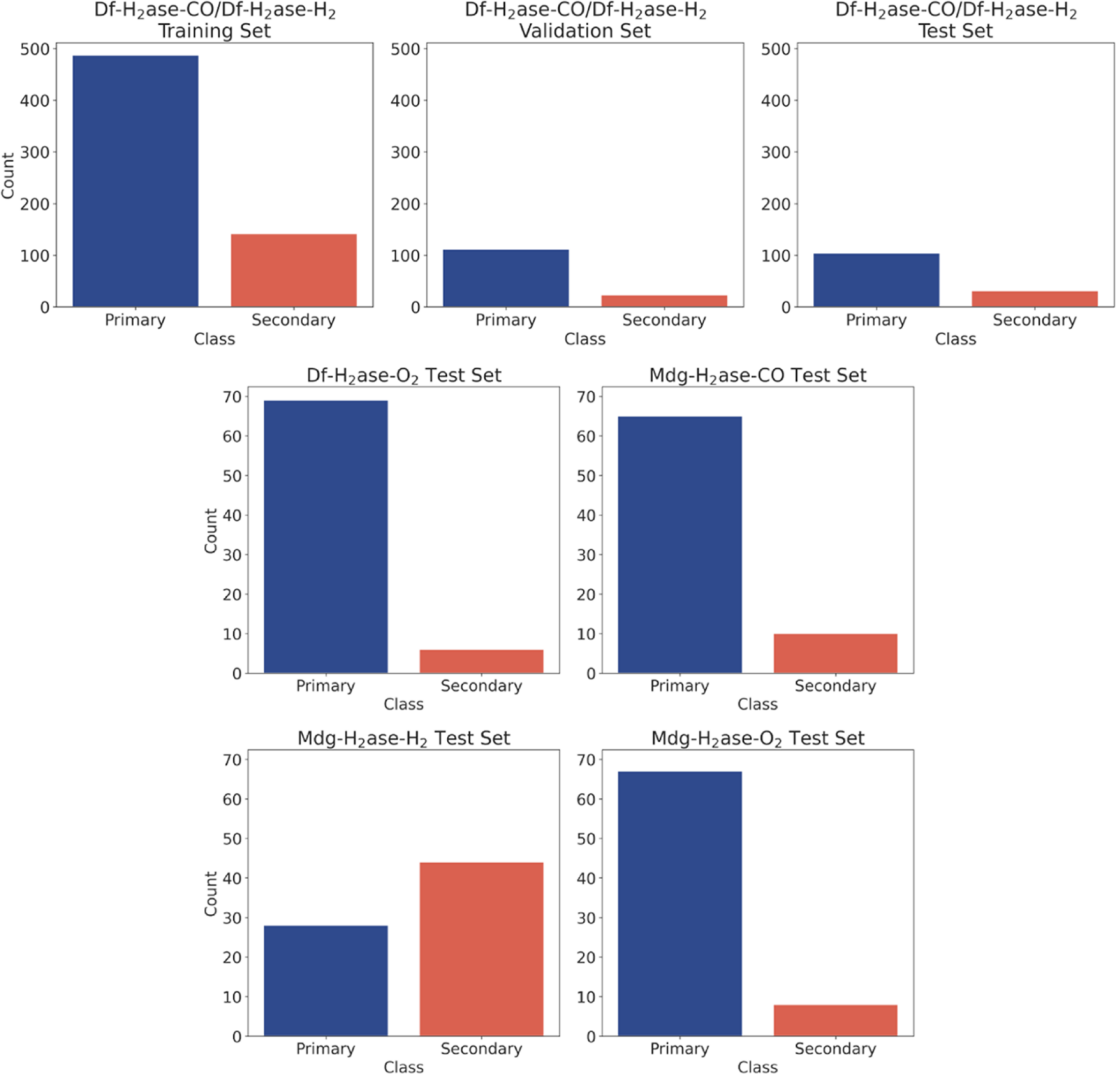
Distribution of the data points in the training, validation and test sets for the binary model. The data sets include CO and H_2_ unbinding from Df H_2_ase (Df-H_2_ase-CO/Df-H_2_ase-H_2_, split into train, validation and test sets), O_2_ unbinding from Df H_2_ase (Df-H_2_ase-O_2_) and CO, H_2_ and O_2_ unbinding from Mdg H_2_ase (Mdg-H_2_ase-CO, Mdg-H_2_ase-H_2_ and Mdg-H_2_ase-O_2_, respectively). The primary class includes tunnels T1, T2 and T7; the secondary class includes tunnels T3-T6, and T8-T10. The data for these distributions are presented in Table S1.

**Figure 3:**
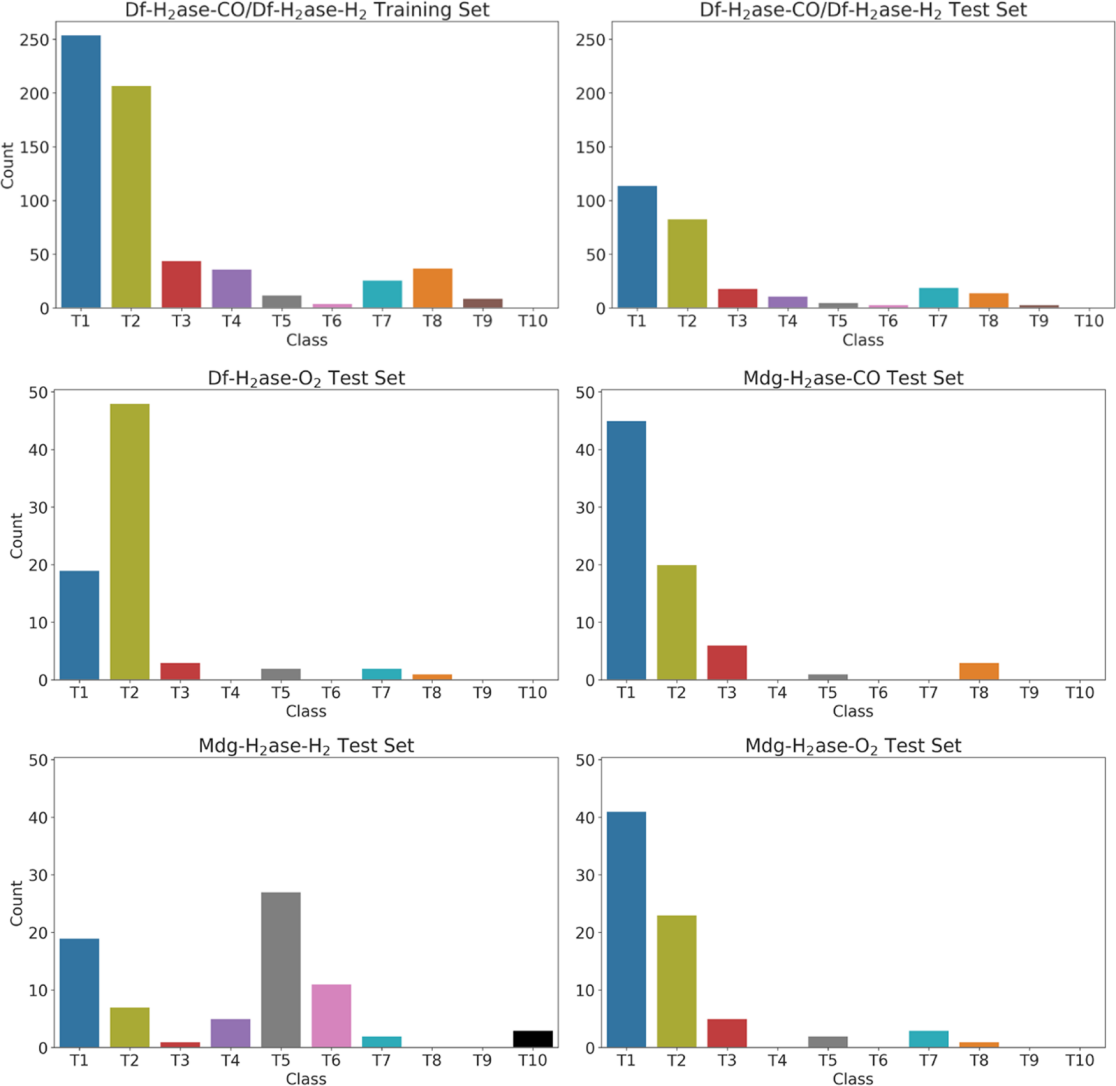
Distribution of the data points in the training and test sets for the multiclass model. The data sets include CO and H_2_ unbinding from Df H_2_ase (Df-H_2_ase-CO/Df-H_2_ase-H_2_, split into train and test sets), O_2_ unbinding from Df H_2_ase (Df-H_2_ase-O_2_) and CO, H_2_ and O_2_ unbinding from Mdg H_2_ase (Mdg-H_2_ase-CO, Mdg-H_2_ase-H_2_ and Mdg-H_2_ase-O_2_, respectively). The colors of each class match the colors of the tunnels in Figure 1. The data for these distributions are presented in Table S2. Optimization of the multiclass model was performed using 5-fold cross-validation. Therefore, no independent validation set was created for this model.

PathInHydro consists of two ML models built based on the data obtained from trajectories. The first ML model is a binary model with two classes: one class (primary) includes the most probable tunnels identified in our previous work,^6^ T1 and T2, together with an adjacent tunnel which shares with T1-T2 the initial part of the unbinding path, T7 (Figure 1); while the other class (secondary) encompasses the rest of the tunnels, all of which are relatively less populated, consisting of T3-T6, T8 and T9. The second ML model is a multiclass model, which treats all of the tunnels separately and has nine classes, T1 to T9. In the analysis of the unbinding events of H_2_ dissociation from Mdg H_2_ase using AQUA-DUCT, we encountered in 3/75 unbinding events a pathway that could not be associated with any of the tunnels identified in Df H_2_ase (Figure S4). This new pathway was subsequently named T10. Since this pathway was not close to the primary pathways and did not share any region with them, we categorized it as one of the secondary pathways. The distribution of the data points in the training and test sets for these two models can be found in Figures 2 and 3 and Tables S1 and S2. There is a strong imbalance in the data sets, where the trajectories belonging to tunnels T1 and T2 tend to hold a significant majority, which led us to use oversampling in the training sets of both the binary and the multiclass models to offset the disproportional class distribution.

**Figure 4:**
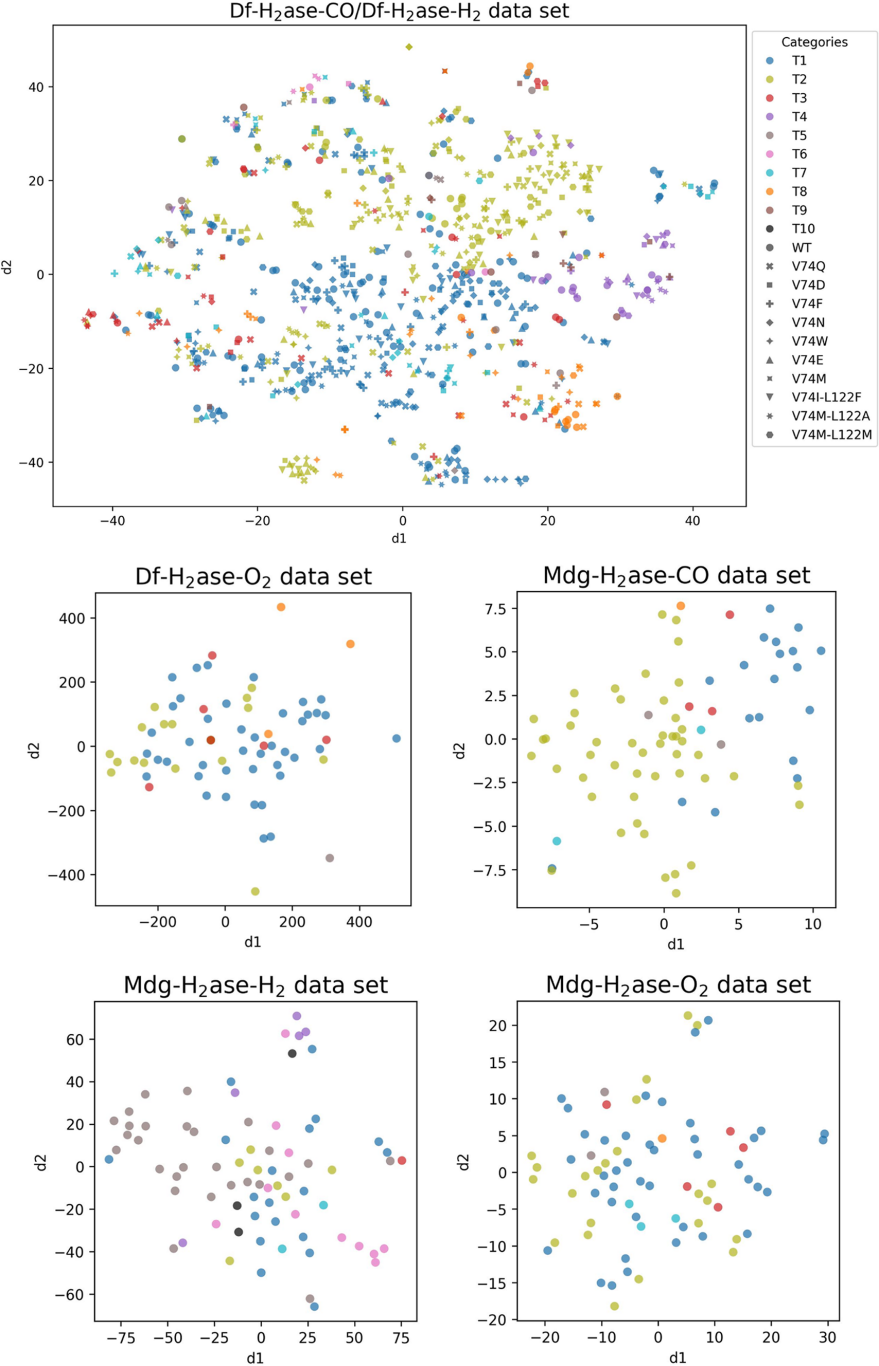
Examination of the data points embedded using the t-SNE algorithm. Data for CO and H_2_ unbinding from Df H_2_ase (Df-H_2_ase-CO/Df-H_2_ase-H_2_), O_2_ unbinding from Df H_2_ase (Df-H_2_ase-O_2_), CO, H_2_ and O_2_ unbinding from Mdg H_2_ase (Mdg-H_2_ase-CO, Mdg-H_2_ase-H_2_ and Mdg-H_2_ase-O_2_, respectively). Every color represents a path, and every symbol represents a mutant. The colors of each path match the colors of the tunnels in Figure 1.

To visually assess the distribution of the data points, we used the t-SNE algorithm,^48^ which transforms high-dimensional data sets into a graphical representation of data structures, arranged based on the similarities among data points. With t-SNE being an unsupervised algorithm, we compared its results with the labels of unbinding paths assigned with the help of AQUA-DUCT. Although the clusters from the t-SNE distribution are relatively loose, and enzyme mutations observably affect the clustering behavior, visual inspection suggests that the local clusters have reasonable levels of consistency in regard to the assigned unbinding pathways (Figure 4). This result led us to reason that, ultimately, this data set is classifiable with the prepared contact time features.

### Performance of the Machine Learning Models

To train the ML models for PathInHydro, we used 70% of the trajectories of CO and H_2_ unbinding from Df H_2_ase (Df-H_2_ase-CO/Df-H_2_ase-H_2_) as training data. As test sets, a subset of Df-H_2_ase-CO/Df-H_2_ase-H_2_ (15% for the binary model, 30% for the multiclass model), as well as the entire collections of simulated unbinding events in Df-H_2_ase-O_2_, Mdg-H_2_ase-CO, Mdg-H_2_ase-H_2_ and Mdg-H_2_ase-O_2_ were used. The model performance on these test sets exemplifies how well the ML models can be generalized to other gas molecules and to other hydrogenases.

Table 1 summarizes the performance of both models for the training and test sets, along with the metrics of the control group, consisting of a baseline model where all entries are assigned to the most populated class. For both models, BA and MCC values close to 1 were achieved for the training set (Table 1, Figure S5), indicating potential overfitting. These high BA and MCC values were obtained despite our efforts to regularize the models and limit their complexity, such as limiting the maximum depth of trees and introducing cost complexity pruning (CCP) afterward.

**Table 1:** Performance of the Binary and the Multiclass Models of PathInHydro for Different Data Sets^a^

	binary model		multiclass model	
	BA	MCC	BA (STD)	MCC (STD)
training set: Df-H_2_ase-CO and Df-H_2_ase-H_2_	0.97	0.94	0.99	0.96
validation^b^	0.98	0.91	0.98 (0.00)	0.97 (0.00)
test set: Df-H_2_ase-CO and Df-H_2_ase-H_2_	0.87	0.71	0.61	0.74
test set: Df-H_2_ase-O_2_	0.84	0.47	0.73	0.47
test set: Mdg-H_2_ase-CO	0.99	0.85	0.69	0.75
test set: Mdg-H_2_ase-H_2_	0.84	0.67	0.54	0.38
test set: Mdg-H_2_ase-O_2_	0.72	0.41	0.42	0.26
baseline model^c^	0.50	0.00	0.11	0.00

a BA: balanced accuracy, MCC: Matthews correlation coefficient, STD: standard deviation.

b External validation set (Df-H_2_ase-CO/Df-H_2_ase-H_2_) for the binary model; 5-fold cross-validation for the multiclass model.

c All data entries were assigned to the dominant class.

**Figure 5:**
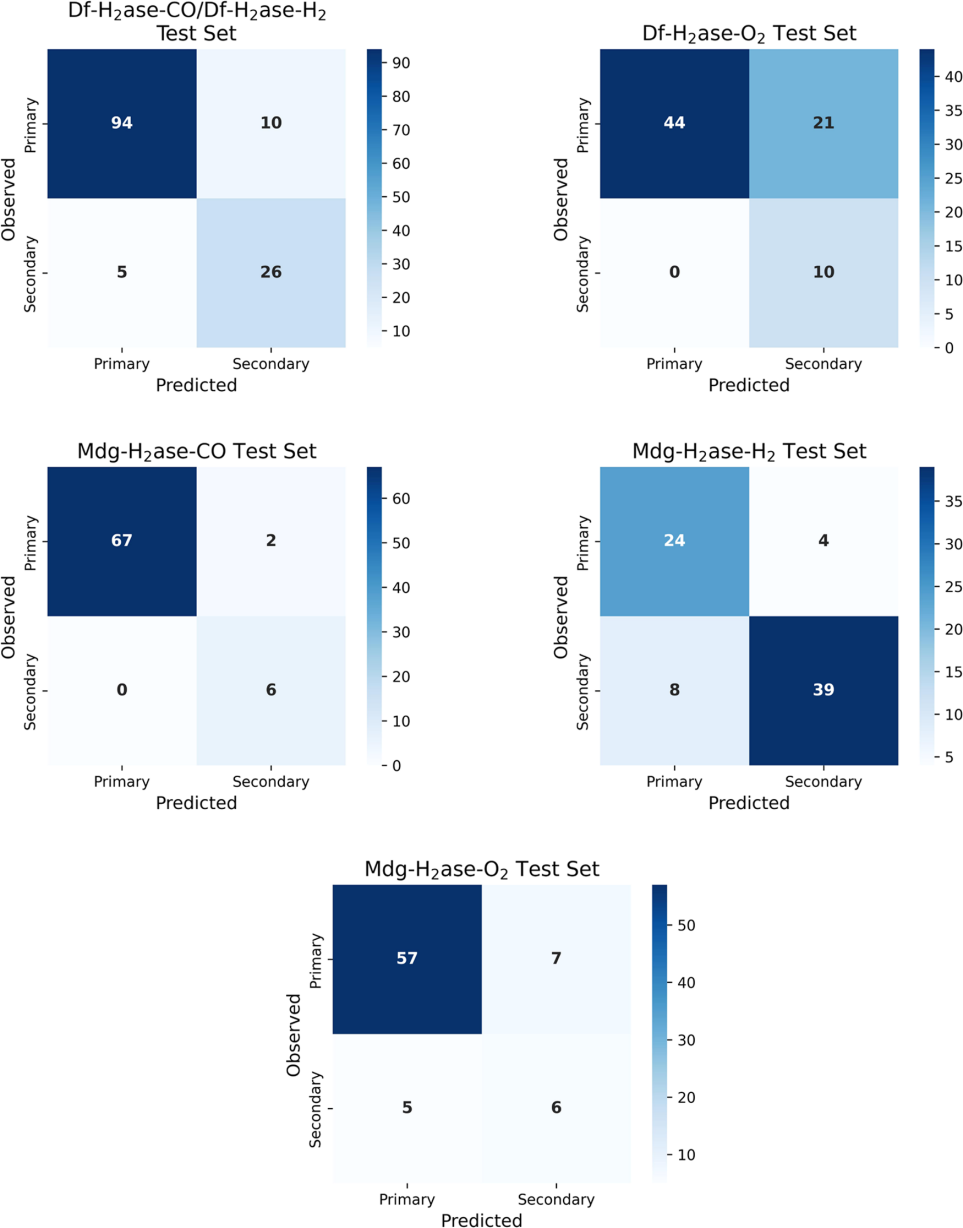
Confusion matrices of the predictions of the binary model of PathInHydro for different test sets: Df-H_2_ase-CO/Df-H_2_ase-H_2_, Df-H_2_ase-O_2_, Mdg-H_2_ase-CO, Mdg-H_2_ase-H_2_ and Mdg-H_2_ase-O_2_. The primary class consists of the T1, T2 and T7 pathways, while the secondary class consists of T3-T6, and T8-T10 pathways.

**Figure 6:**
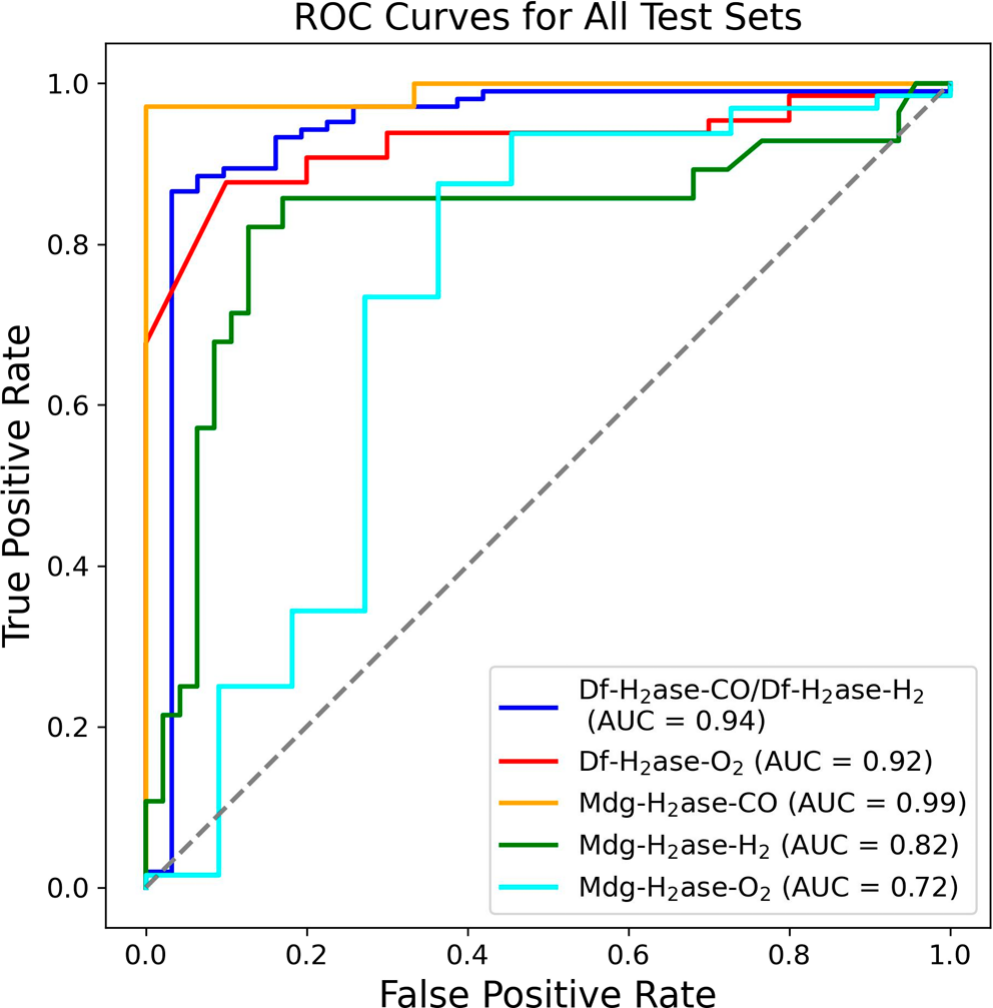
Receiver Operating Characteristic (ROC) curves and area under the curve (AUC) values of the binary model for different test sets (Df-H_2_ase-CO/Df-H_2_ase-H_2_, Df-H_2_ase-O_2_, Mdg-H_2_ase-CO, Mdg-H_2_ase-H_2_ and Mdg-H_2_ase-O_2_).

For the binary model, the BA values and MCC values for the five test sets varied from 0.72 to 0.99 and from 0.41 to 0.85, respectively (Table 1), which are higher than the values for the baseline model, indicating that the model is useful and transferable to different gas molecules and enzymes. Confusion matrices show the detailed breakdown of the predictions for each test set (Figure 5). Notably, in two of the test sets (Df-H_2_ase-O_2_ and Mdg-H_2_ase-CO), no secondary paths were misclassified as primary paths. ROC curves were also employed to examine the binary model (Figure 6), and the high area under the curve (AUC) values (0.72–0.99) for the test sets indicate that the binary model can successfully distinguish between the two classes.

The MCC values for the Df-H_2_ase-O_2_ and Mdg-H_2_ase-O_2_ test sets were conspicuously lower than for the other test sets. We reasoned that either the absence of O_2_ trajectories in the training set, or the larger size of O_2_ may set it too much apart from the other two types of gas molecules tested, leading to a set of features (enzyme-gas interactions) that differs from the features for CO and H_2_, resulting in a difficulty for the model to make accurate predictions for O_2_ pathways. However, we observed that the limitation of the binary model to predict O_2_’s pathways could be rectified using the multiclass model, which will be discussed later.

Taken together, the results show that the binary model can accurately distinguish between the primary and the secondary unbinding pathways for different gas molecules and for different hydrogenases, making it applicable for research projects where one attempts to add blockage to the primary tunnels, thus regulating the passage of larger gas molecules.

**Figure 7:**
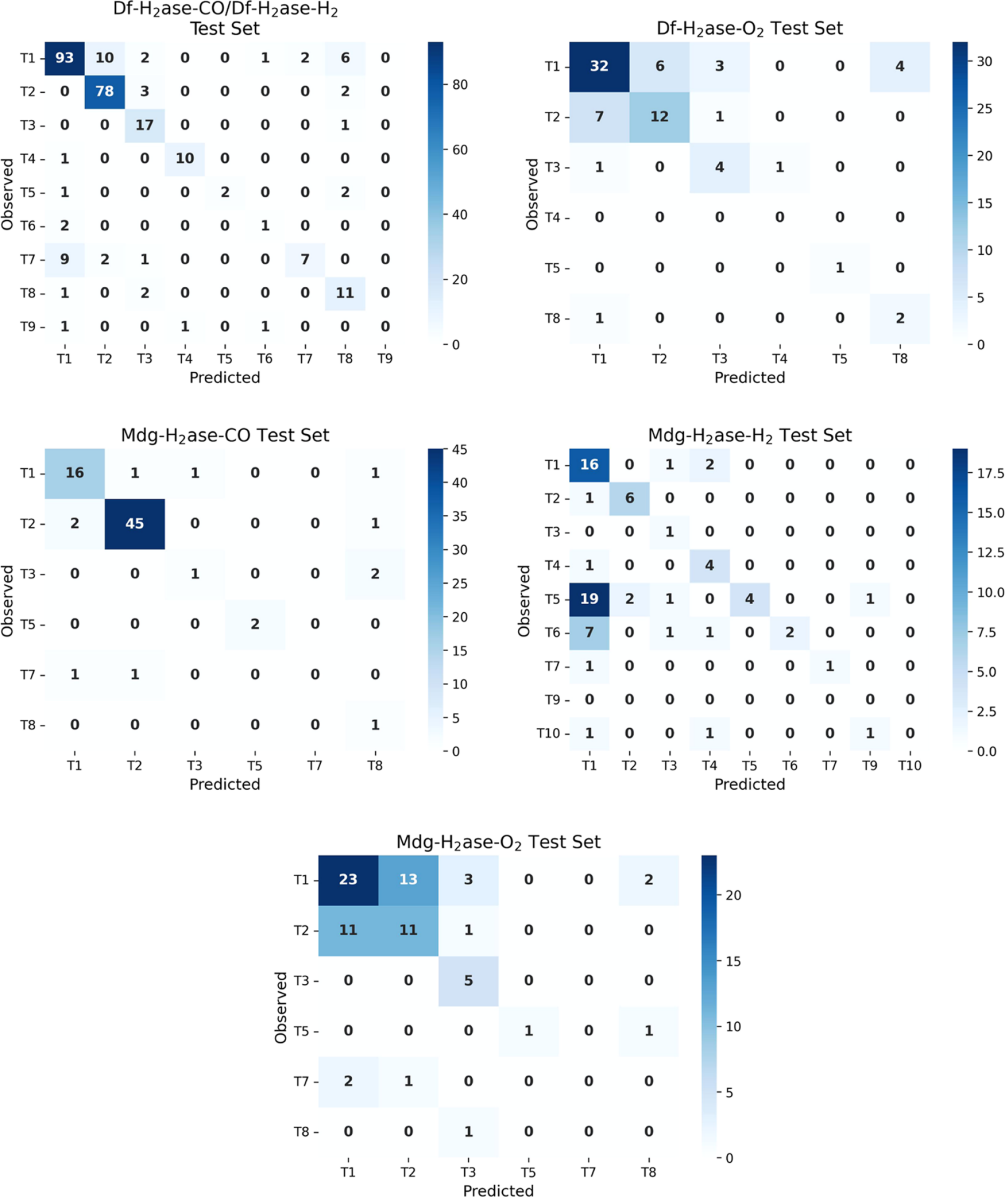
Confusion matrices of the predictions of the multiclass model of PathInHydro for different test sets: Df-H_2_ase-CO/Df-H_2_ase-H_2_, Df-H_2_ase-O_2_, Mdg-H_2_ase-CO, Mdg-H_2_ase-H_2_ and Mdg-H_2_ase-O_2_. Some paths are absent in some test sets.

For the multiclass model, the BA values and MCC values for the four test sets varied from 0.42 to 0.73 and from 0.26 to 0.75, respectively (Table 1). The BA and MCC values for the test sets are higher than the values for the baseline model, indicating that the model is useful and transferable for different gas molecules and enzymes. In the multiclass model, the BA and MCC values for the test sets are generally lower in comparison to the binary model. The drop in performance was expected, due to the higher number of classes to distinguish, which increases the possibility of misassignments. Furthermore, the elevated imbalance in class distribution makes it hard to estimate the potential assignment accuracy for the less populated pathways, the most scarce of which contain only 1 or 2 data points (Figure 7, Table S2). Path T10, which only appeared in the Mdg-H_2_ase-H_2_ test set, could not be classified correctly, since it was not present in the training set.

The confusion matrices (Figure 7) show that a considerable portion of the errors comes from misclassification of various trajectories as the majority class T1, while the misassignment of true T1 trajectories is relatively high in number, but low in proportion. Intriguingly, a large fraction of the misassignments in the multiclass model are internal, meaning that trajectories belonging to one primary pathway are misassigned as another primary pathway, and the same for secondary pathway trajectories. For example, in the Df-H_2_ase-CO/Df-H_2_ase-H_2_ test set (Figure 7), misclassified T1 pathways are mostly assigned as the two other primary pathways T2 and T7. Additionally, we identified the proximity of the pathway exit points to each other as another important contributor to misclassification. As an example, T3 is located between T8 and T1 in the structure of Df H_2_ase (Figure 1), and as presented in Figure 7, misclassified T3 pathways are often assigned as T1 or T8 pathways. Furthermore, we also observed that the multiclass model has fewer external misclassifications between the primary and secondary pathways. Therefore, we used the multiclass model to perform binary classification (Table 2 and Figure S6), and the results were mostly similar to the binary model presented in Table 1. However, with the multiclass model, we observed an improved performance in predicting O_2_’s pathways in both Df and Mdg H_2_ase, in comparison to the binary model. This also speaks favorably of the accuracy and transferability of the multiclass model.

**Table 2:** Performance of the Multiclass Model in the Prediction of Binary Pathways^a^

	BA	MCC
training set: Df-H_2_ase-CO and Df-H_2_ase-H_2_	0.99	0.96
test set: Df-H_2_ase-CO and Df-H_2_ase-H_2_	0.91	0.77
test set: Df-H_2_ase-O_2_	0.84	0.56
test set: Mdg-H_2_ase-CO	0.98	0.80
test set: Mdg-H_2_ase-H_2_	0.63	0.28
test set: Mdg-H_2_ase-O_2_	0.96	0.72

a BA: balanced accuracy, MCC: Matthews correlation coefficient.

Overall, the results suggest the viability of the multiclass model for the automatic preliminary assignment of unbinding pathways for a variety of gas molecules, and with tolerance toward different types of H_2_ases.

We quantified feature importance in both models using mean decrease in impurity to investigate which ligand-residue contacts are key features for path classification (Figures S7 and S8). The importance values were low, probably due to the high number of features (804). We found that the contacts with residues surrounding the exit points of paths tend to have relatively high importance, since these regions are exclusive to each pathway. For example in Figure S7 we can see that residue V273 from the large subunit has the highest importance, and it is located at the exit of T3. Moreover, the residue with the second highest importance was L167 from the large subunit, which is located at the exit of T1. L162, another residue with high importance, is located at the exit of T2.

## Discussion

Prior to PathInHydro, several methods available in the literature were capable of identifying unbinding pathways in trajectories from MD simulations, using residue-ligand contacts, or the ligand's distances to tunnel bottlenecks or protein residues, in an unsupervised way. In comparison, PathInHydro is a supervised, pretrained classification model which relies on existing labels acquired from tracking the gas molecules in unbinding trajectories by AQUA-DUCT and visual inspection. While this can be considered a limitation, the goal of this set of models is to sort data into established frameworks promptly. Since PathInHydro is pretrained, it enables speedy classification of (un)binding pathways. Moreover, while many of the methods presented in the introduction can be applied to any protein, PathInHydro is restricted to hydrogenases, since this is the enzyme used to train the models.

To further investigate the generalizability of PathInHydro to other hydrogenases, we calculated sequence similarity and identity of O_2_-tolerant and O_2_-sensitive hydrogenases using Df H_2_ase, the enzyme used in the training set, as a reference (Table S5). We considered all experimental structures for soluble [NiFe] hydrogenases listed in Table 9 of ref (27). Our MD simulations were restricted to O_2_-sensitive enzymes, such as Df H_2_ase and Mdg H_2_ase, because there are no force field parameters available for all metal centers of O_2_-tolerant enzymes. As can be seen in Table S5, Df H_2_ase and Mdg H_2_ase are very similar (67 and 64% sequence identity for the small and large subunits, respectively), while there is less sequence identity between Df H_2_ase and O_2_-tolerant H_2_ases. Given the high sequence identity between Df H_2_ase and other O_2_-sensitive H_2_ases, and the good performance of the model for Mdg H_2_ase, we expect PathInHydro to generalize well for other O_2_-sensitive H_2_ases. Further tests, however, are required to see if PathInHydro can also be used for O_2_-tolerant H_2_ases. To follow up on this study, we plan to develop force field parameters for the metal centers of O_2_-tolerant H_2_ases, then perform MD simulations for gas unbinding and test PathInHydro in the identification of unbinding paths for these enzymes.

Regarding generalizability to other gas molecules and ligands, in our study we were concerned with the air resistance of H_2_ase, so the gas molecules of interest were substrates and inhibitors with relevant concentrations in the air, which in this case may also include NO. While NO was not included in our current simulations, we believe NO is sufficiently similar to CO and O_2_ for its behavior to be classified with reasonable accuracy. Additionally, training a supervised model to classify unbinding paths for small gas molecules is challenging due to the many unbinding pathways available. We predict that training similar models may be less challenging for enzymes and proteins that bind to larger molecules, since these systems usually have a lower diversity of unbinding paths.

It is important to mention that there are limitations associated with the ML models of PathInHydro. First, they can only be used for [NiFe] hydrogenases, since the unbinding pathways and associated features that the ML models were trained on are specific to this enzyme. Second, these supervised models are unable to recognize new pathways that are not present in the training set. This can pose a hindrance to their generalizability for mutant forms of [NiFe] hydrogenase, where new tunnels and pathways may be available. This is exemplified in our work, where we have encountered a new unbinding pathway for H_2_ dissociation from Mdg H_2_ase, path T10, that could not be associated with any of the Df H_2_ase tunnels. Despite the shortcomings, we believe that these models are a proof of concept that protein–ligand contact data derived from MD simulations can be used to build supervised ML models that are capable of identifying unbinding pathways.

The initial identification of unbinding pathways in the trajectories used to train the models was based on visual inspection to match the tunnels identified using CAVER with the pathways identified by AQUA-DUCT in simulations of ligand unbinding in different enzymes and mutants. Visual inspection is susceptible to subjectivity and human error. In this sense, the ML models provided here can assist in the identification of possible inconsistencies.

Another major challenge in pathway classification is that the unbinding trajectories of ligands are not always straightforward, and the ligand can visit large portions of multiple tunnels prior to achieving the unbound state. In some trajectories, once the small molecule leaves the bound state, it can roam in the network of tunnels inside the protein and visit different tunnels before eventually unbinding. This behavior was also observed for larger substrate molecules, as reported by Sarkar et al.,^22^ who proposed a tunnel classification scheme to deal with such events. This means that, in some cases, large segments of the trajectory can be considered noise in the data, since such trajectories will contain contacts that characterize more than one tunnel, which can hamper the performance of the ML models in the identification of pathways. Overall, we believe there is room for improvements on both data gathering from unbinding trajectories, and feature engineering to better represent the unbinding process, which we will address in future work.

## Conclusions

Nowadays, with the availability of high performance computing and graphics processing units to speed up MD simulations, it is possible to run a large number of simulations to compute kinetic rates and pathway probabilities for protein–ligand dissociation on a statistically meaningful level. The large number of trajectories, however, poses a problem for data analysis and pathway assignment, if manual inspection is employed in the process. Therefore, ML models capable of identifying unbinding pathways in simulations can be a valuable tool to speed up data analysis.

In this work, we have presented PathInHydro, a set of two supervised ML models, one binary and one multiclass, to assign unbinding paths for the dissociation of gas molecules from [NiFe] hydrogenases, using the contacts between the ligand and the residues of the protein as features. The binary model identifies primary (most probable) and secondary (less probable) paths, while the multiclass model delineates 9 paths. The models were trained on data for CO and H_2_ dissociation from Df H_2_ase only, and we were able to demonstrate the generalizability of this approach, showing that these models are applicable for other gas molecules, such as O_2_, and for other hydrogenases, such as Mdg H_2_ase. While both ML models have better performance than the baseline models, one can see that the binary assignment is more accurate, while the multiclass model should be used with care. We believe that PathInHydro can assist in the automated identification of unbinding pathways of gas molecules from H_2_ases in simulations, facilitating and speeding up data analysis when many trajectories have to be evaluated.

## Data Availability

The code (jupyter notebooks) and the data sets used to train and test the ML models are available online at: https://github.com/FarzinSohraby/PathInHydro.

## Supporting Information Available

Additional information about the methods and results (PDF)
