# Supplementary material for: PathInHydro, a Set of Machine Learning Models to Identify Unbinding Pathways of Gas Molecules in [NiFe] Hydrogenases
Source: J Chem Inf Model. 2025 Jan 7;65(2):589–602. doi: 10.1021/acs.jcim.4c01656 (PMC11776054; doi:10.1021/acs.jcim.4c01656)
Supplement: Supplementary file 1 — ci4c01656_si_001.pdf [file ci4c01656_si_001.pdf]

Supporting Information

PathInHydro, a set of machine learning models to identify unbinding pathways of gas molecules in [NiFe] hydrogenases

Farzin Sohraby, Jing-Yao Guo, Ariane Nunes-Alves*

Institute of Chemistry, Technische Universität Berlin, Straße des 17. Juni 135, 10623 Berlin, Germany

*Corresponding author: ferreira.nunes.alves@tu-berlin.de

Structural similarity between the [NiFe] hydrogenases from *Desulfovibrio fructosovorans* and from *Megalodesulfovibrio gigas*

As a test set to investigate if the machine learning (ML) models can predict the unbinding pathways of CO, H_2 and O_2 for a hydrogenase similar to the [NiFe] hydrogenase (H_2ase) from *Desulfovibrio fructosovorans* (Df H_2ase), we considered the *Megalodesulfovibrio gigas* H_2ase (Mdg H_2ase) (PDB ID 1YQ9[1]) and simulated the pathways for CO, H_2 and O_2 unbinding from the catalytic site using molecular dynamics (MD) simulations and τRAMD with the same parameters previously used for the simulations of the Df H_2ase (PDB ID 1YQW[1]). The two enzymes were structurally aligned using UCSF chimera[2] and the backbone root mean square deviation (RMSD) value calculated was 0.6 Å (Figure S1). The structural alignment was used for the matching of the residues of the two enzymes (Figures S2 and S3), which was a prerequisite for the protein-ligand contacts to be used as features in the ML models.

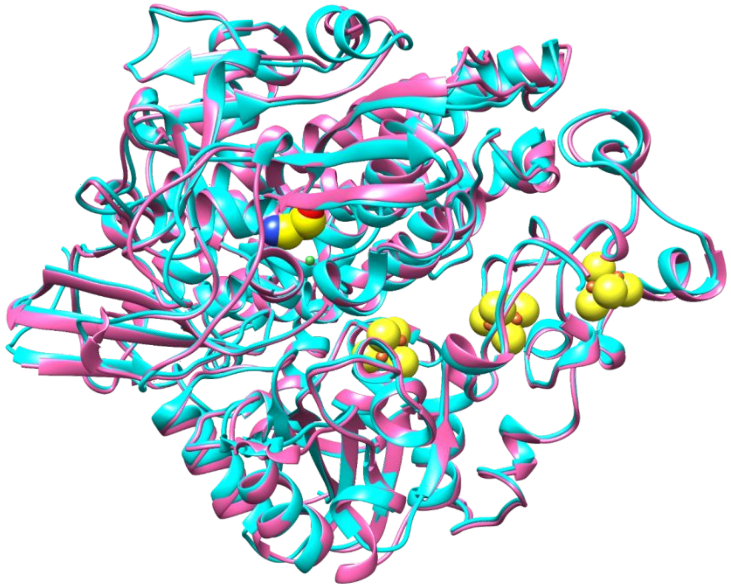

Figure S1. Structural alignment of Df H_2ase (cyan) and Mdg H_2ase (pink), with a backbone RMSD value of 0.6 Å calculated by UCSF chimera software[2]. Colored spheres indicate the positions of the metal centers.

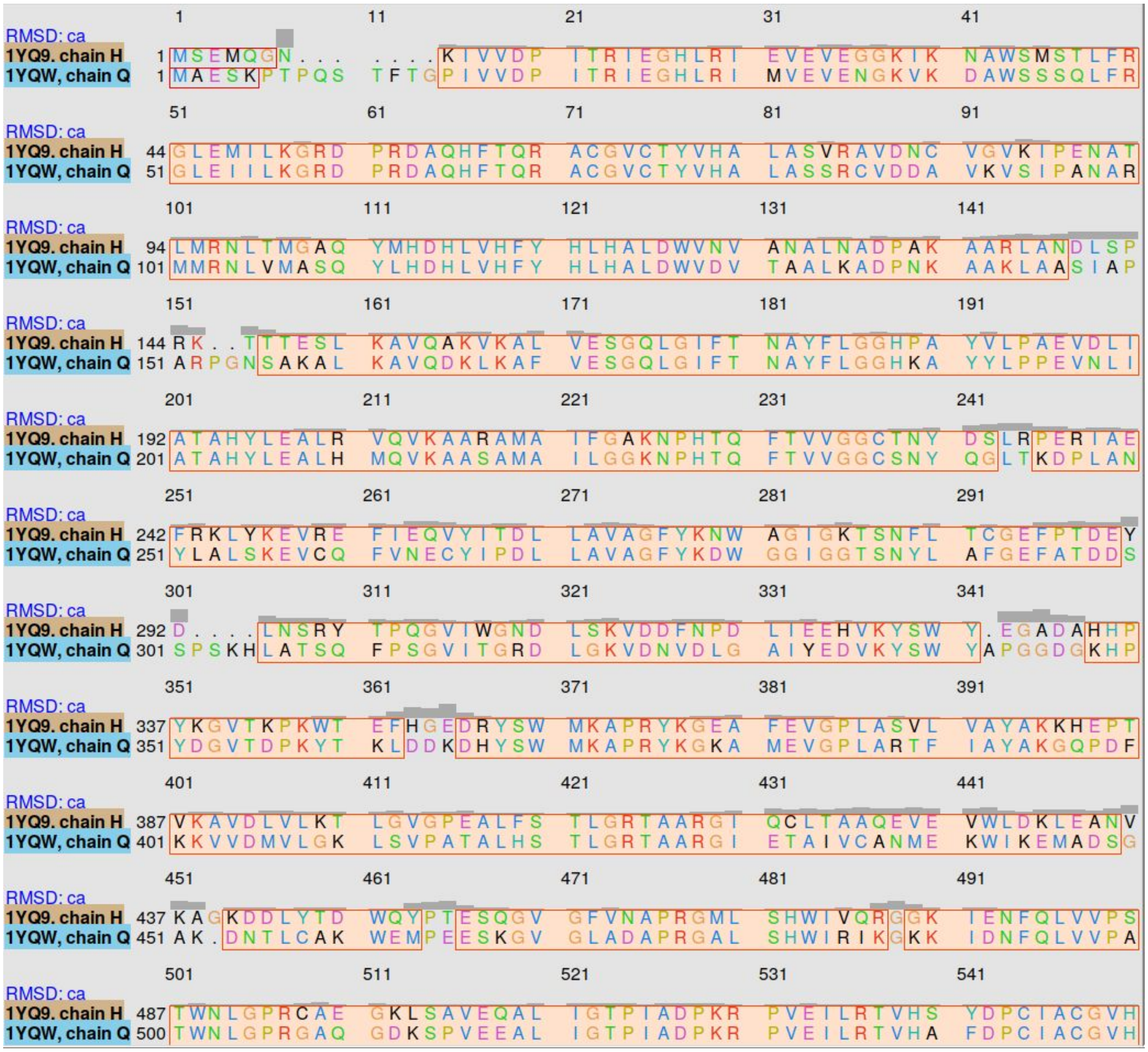

Figure S2. Sequence alignment of the large subunits of Df H_2ase (PDB ID 1YQW[1]) and Mdg H_2ase (PDB ID 1YQ9[1]) based on structural alignment. The analysis and the image were produced by UCSF chimera.

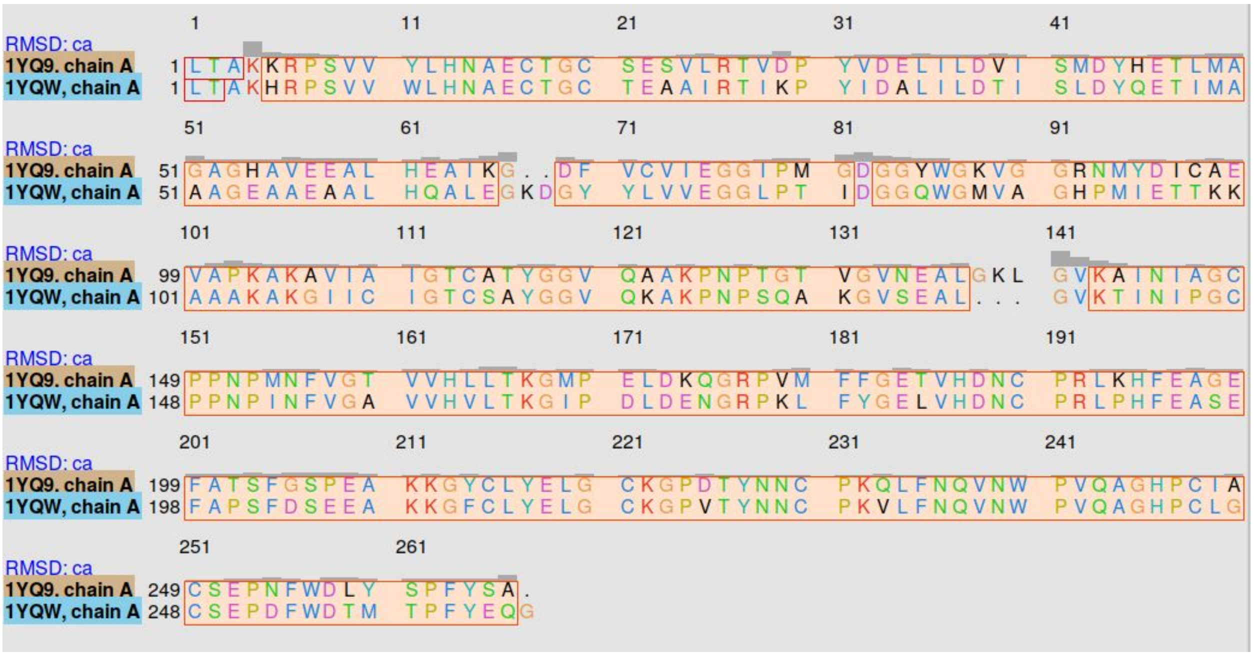

Figure S3. Sequence alignment of the small subunits of Df H_2ase (PDB ID 1YQW[1]) and Mdg H_2ase (PDB ID 1YQ9[1]) based on structural alignment. The analysis and the image were produced by UCSF chimera[2].

Distribution of the data points used to train the binary and multiclass models

Table S1. Distribution of the data points in the binary data set.

Class*	Df-H_2ase-CO/Df-H_2ase-H_2 (training set)	Df-H_2ase-CO/Df-H_2ase-H_2 (validation set)	Df-H_2ase-CO/Df-H_2ase-H_2 (test set)	Df-H_2ase-O_2 (test set)	Mdg-H_2ase-CO (test set)	Mdg-H_2ase-H_2 (test set)	Mdg-H_2ase-O_2 (test set)
Primary	487	112	104	65	69	28	67
Secondary	142	23	31	10	6	47	8

*T1, T2 and T7 are considered primary pathways, and T3-T6, T8-T10 are considered secondary pathways.

Table S2. Distribution of the data points in the multiclass data set.

Class	Df-H_2ase-CO/Df-H_2ase-H_2 (training set)	Df-H_2ase-CO/Df-H_2ase-H_2 (test set)	Df-H_2ase-O_2 (test set)	Mdg-H_2ase-CO (test set)	Mdg-H_2ase-H_2 (test set)	Mdg-H_2ase-O_2 (test set)
T1	254	114	45	19	19	41
T2	207	83	20	48	7	23
T3	44	18	6	3	1	5
T4	36	11	0	0	5	0
T5	12	5	1	2	27	2
T6	4	3	0	0	11	0
T7	26	19	0	2	2	3
T8	37	14	3	1	0	1
T9	9	3	0	0	0	0
T10	0	0	0	0	3	0

Test of different machine learning classification algorithms for the binary and the multiclass models

We tested different algorithms for the classification tasks. The GridsearchCV method from scikit-learn[3] was used to find the best hyperparameter values. The best hyperparameter values found for each algorithm for the binary model are as follows:

- **Random Forest**: a ccp_alpha of 0, a maximum depth of 10, a maximum sample size of 0.7, 500 estimators.
- **Adaptive Boosting (AdaBoost)**: a learning rate of 0.05, 500 estimators.
- **Gradient Boosting**: a learning rate of 0.01, a maximum depth of 3 with 500 estimators, and a subsample size of 0.8.
- **Support vector machine (SVM)**: a regularization parameter C of 100, gamma value set to "scale", and the radial basis function (RBF) kernel.
- **Logistic Regression**: pure L2 regularization with a strength C of 10, and "newton-cg" was used as the solver.
- **k-nearest neighbors (kNN)**: a k of 9, with distance-based weighting, and the Manhattan distance metric.

The AdaBoost algorithm was selected for the binary model, since it provided the best performance for the validation set (Table S3).

The best hyperparameter values found for each algorithm for the multiclass model are as follows:

- **Random Forest**: a ccp_alpha of 0, a maximum depth of 30, a maximum sample size of 0.95 and with 500 estimators.
- **AdaBoost**: a learning rate of 0.5 with 2000 estimators.
- **Gradient Boosting**: a learning rate of 0.05, a maximum depth of 3, with 500 estimators and a subsample size of 0.7.
- **Support vector machine (SVM)**: a regularization parameter C of 100, gamma value set to "scale" and the RBF kernel.
- **Logistic Regression**: pure L2 regularization with a strength C of 100, with "saga" used as the solver.
- **k-nearest neighbors (kNN)**: a k of 3, with distance-based weighting, and the Manhattan distance metric.

The random forest algorithm was selected for the multiclass model, since it is a robust algorithm and is among the best performers for the validation set. We changed the hyperparameters for the model from table S4 slightly to introduce regularization to the model without impeding its performance.

Table S3. Validation performances of different classification algorithms for the binary models. BA: balanced accuracy, MCC: Matthews correlation coefficient.

**Algorithm**	**BA**	**MCC**
AdaBoost	0.98	0.91
Gradient Boosting	0.96	0.86
Random Forest	0.96	0.86
Logistic Regression	0.90	0.76
SVM[a]	0.89	0.71

kNN[b]	0.86	0.63

[a]SVM: support vector machine

[b]kNN: k-Nearest Neighbors

Table S4. Performance for the validation set of different classification algorithms to build the multiclass models. BA: balanced accuracy, MCC: Matthews correlation coefficient.

**Algorithm**	**BA**	**MCC**
Gradient Boosting	0.98	0.98
Random Forest	0.98	0.97
SVM[a]	0.95	0.95
Logistic Regression	0.94	0.94
kNN[b]	0.93	0.92
AdaBoost	0.78	0.76

[a]SVM: support vector machine

[b]kNN: k-Nearest Neighbors

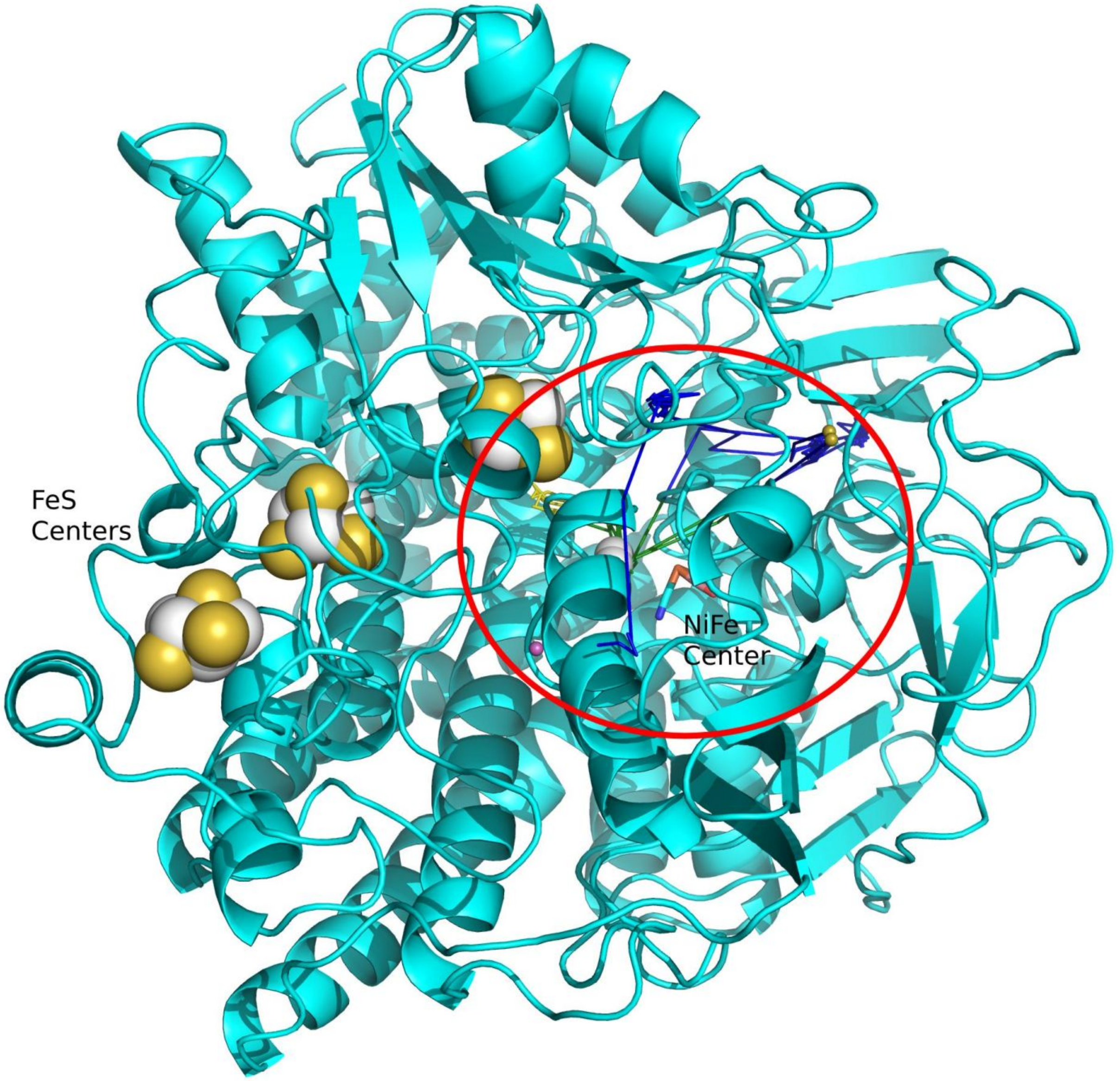

Figure S4. Location of the pathway T10, found in the simulations of H_2 dissociation from Mdg H_2ase. The red circle highlights the location of the H_2 exit points in the three unbinding trajectories attributed to T10, shown as spheres. The blue traces show the unbinding route.

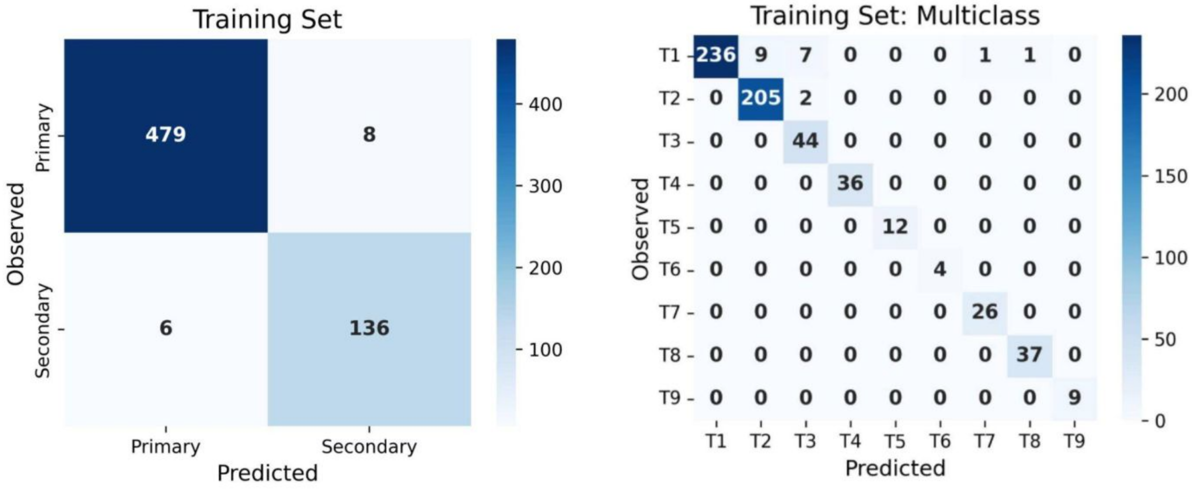

Figure S5. Confusion matrices of the binary and the multiclass models for the Df-H_2ase-CO/Df-H_2ase-H_2 training set.

Using the multiclass model to perform binary classification

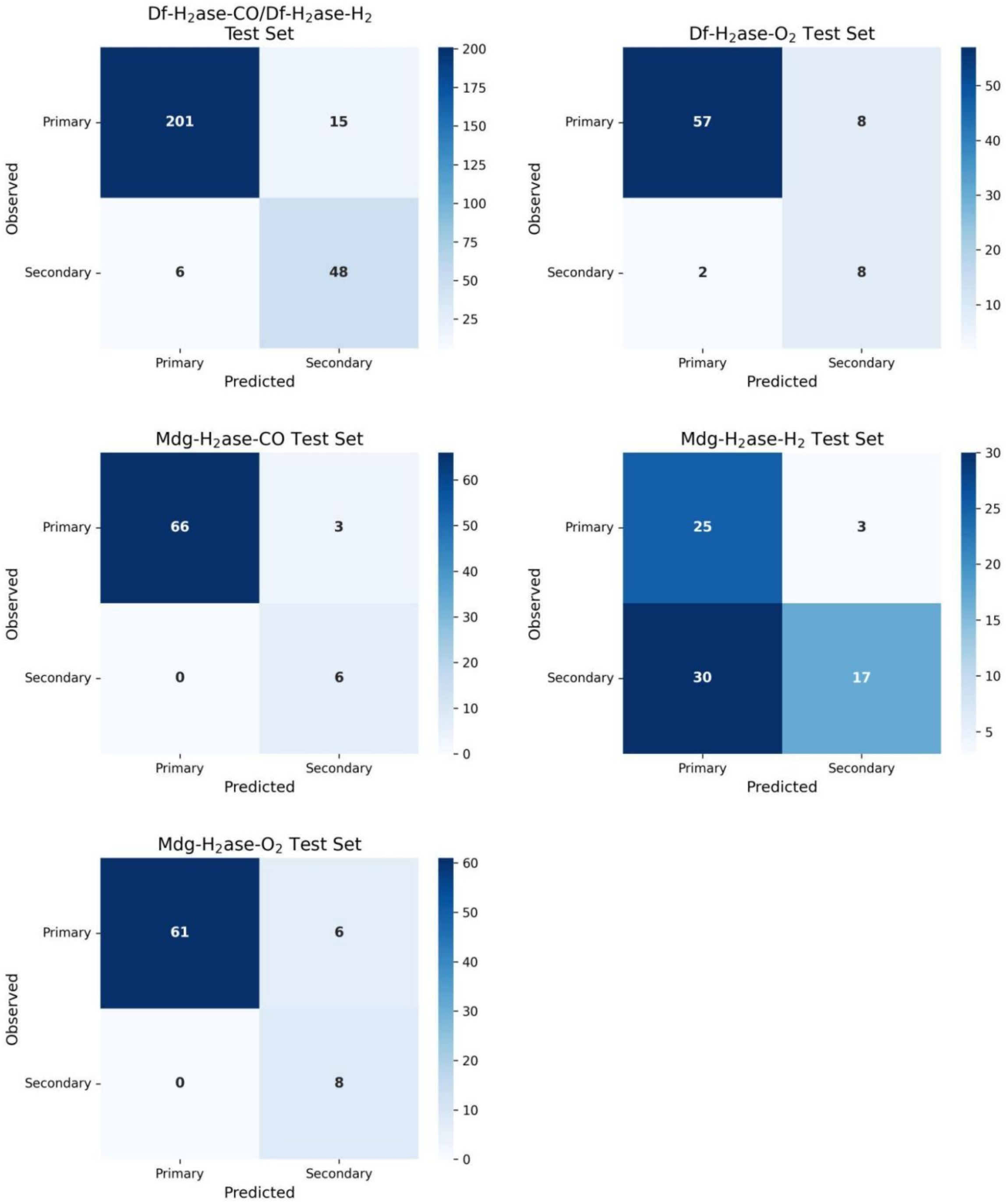

Figure S6. Confusion matrix for the prediction of the binary classes using the multiclass model.

Table S5. Sequence similarity and identity of the large and small subunits of a number of different soluble [NiFe] H_2ases compared to the Df H_2ase. FF: force field. Experimental structures obtained from table 9 of ref. [4].

H_2ase (PDB)	Organism	Seq. similarity - small subunit	Seq. similarity - large subunit	Seq. identity - small subunit	Seq. identity - large subunit	complete FF available?	O_2-tolerant?
1YQ9	D. gigas (Mdg)	78.7%	73.9%	67.0%	64.1%	yes	no
1UBH	D. vulgaris MF	76.9%	74.9%	63.8%	64.4%	yes	no
4C3O	S. enterica	60.7%	61.0%	45.0%	44.8%	no	yes
3USE	E. coli	50.0%	57.5%	36.3%	42.8%	no	yes
7UUR	M. smegmatis	38.9%	40.5%	23.6%	27.6%	no	yes

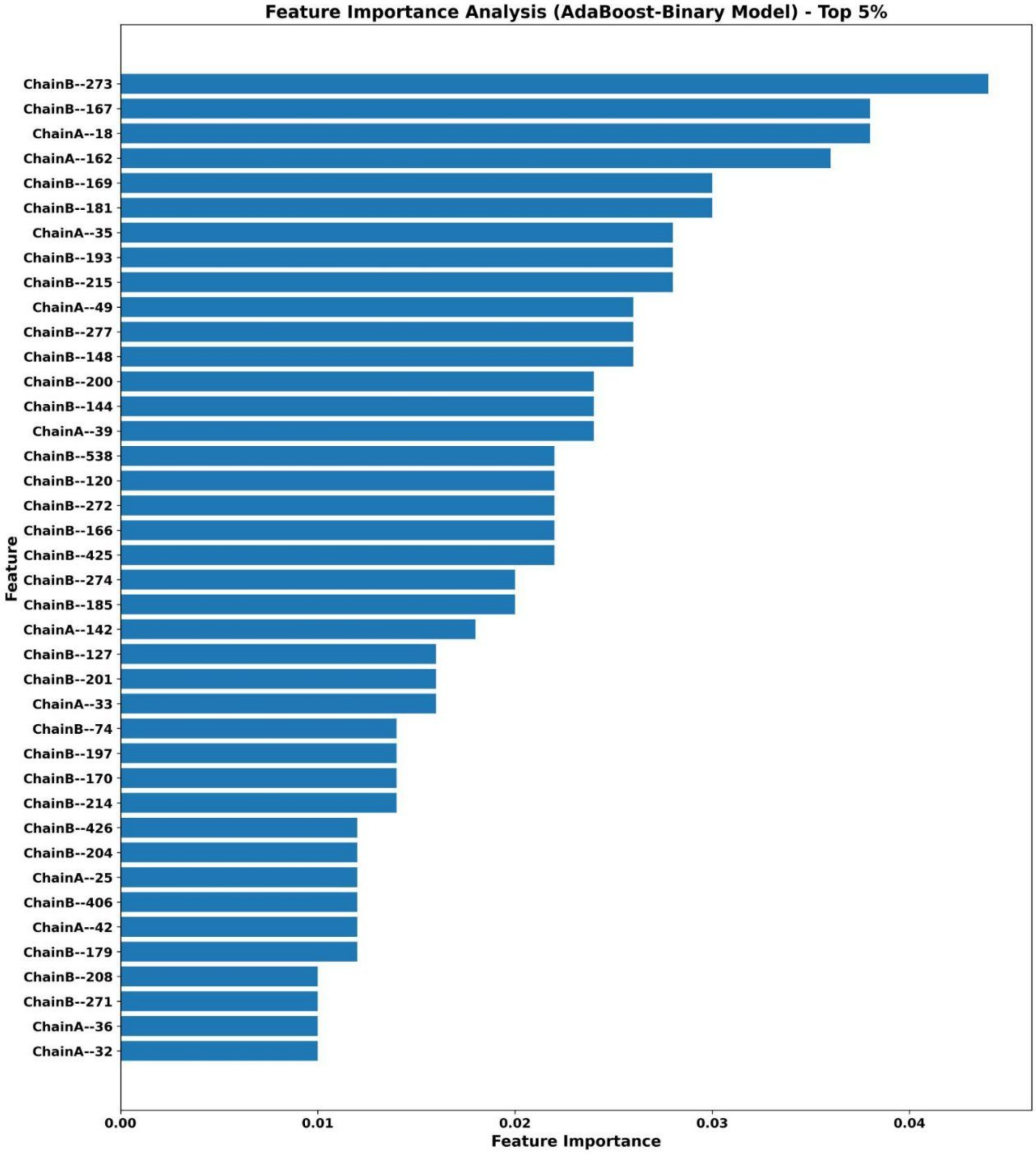

Figure S7. Feature importance of the binary model, calculated as the mean decrease in impurity, also known as Gini importance. Only the top 5% features are shown. Chain A is the small subunit and chain B is the large subunit.

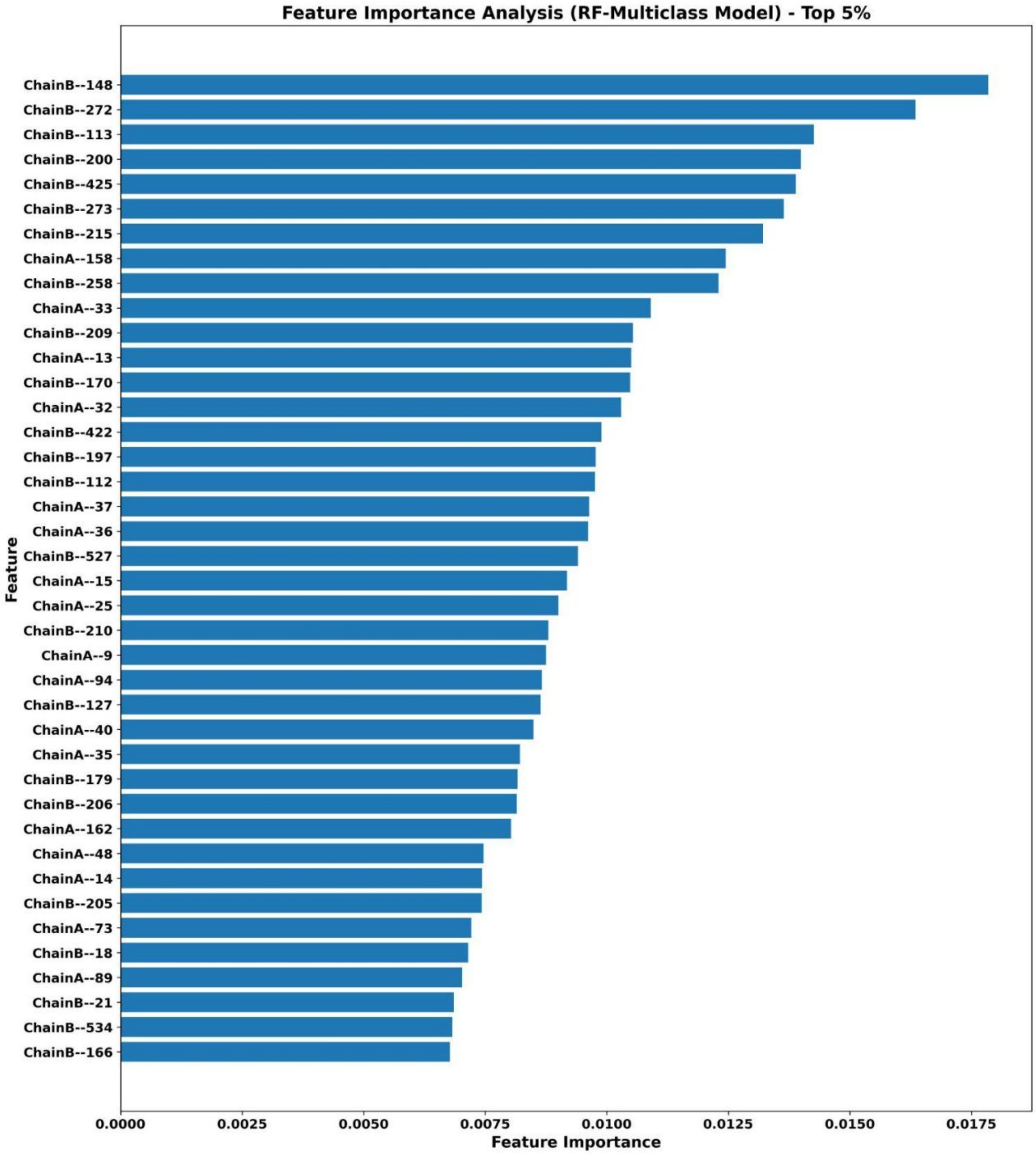

Figure S8. Feature importance of the multiclass model, calculated as the mean decrease in impurity, also known as Gini importance. Only the top 5% features are shown. Chain A is the

small subunit and chain B is the large subunit.

References

(1) Volbeda, A.; Martin, L.; Cavazza, C.; Matho, M.; Faber, B. W.; Roseboom, W.; Albracht, S. P. J.; Garcin, E.; Rousset, M.; Fontecilla-Camps, J. C. Structural Differences between the Ready and Unready Oxidized States of [NiFe] Hydrogenases. *J Biol Inorg Chem* **2005**, *10* (3), 239–249. https://doi.org/10.1007/s00775-005-0632-x.

(2) Pettersen, E. F.; Goddard, T. D.; Huang, C. C.; Couch, G. S.; Greenblatt, D. M.; Meng, E. C.; Ferrin, T. E. UCSF Chimera?A Visualization System for Exploratory Research and Analysis. *J. Comput. Chem.* **2004**, *25* (13), 1605–1612. https://doi.org/10.1002/jcc.20084.

(3) Pedregosa, F.; Varoquaux, G.; Gramfort, A.; Michel, V.; Thirion, B.; Grisel, O.; Blondel, M.; Prettenhofer, P.; Weiss, R.; Dubourg, V.; Vanderplas, J.; Passos, A.; Cournapeau, D.; Brucher, M.; Perrot, M.; Duchesnay, É. Scikit-Learn: Machine Learning in Python. *J. Mach. Learn. Res.* **2011**, *12* (null), 2825–2830.

(4) Lubitz, W.; Ogata, H.; Rüdiger, O.; Reijerse, E. Hydrogenases. *Chem. Rev.* **2014**, *114* (8), 4081–4148. https://doi.org/10.1021/cr4005814.
